# 

**Published:** 1892-03

**Authors:** 


### CONTENTS FOR MARCH, 1892.

ORIGINAL COMMUNICATIONS.

Nerve Counterfeits of Uterine Disease. By HENRY D. INGRAHAM, M. D. .... 449
Senile Gangrene. By HERMAN MYNTER, M. D. .... 457
The Faradic Coil in Gynecological Practice. By HERMAN E. HAYD, M. D. .... 465
A Retrospect of Medical Progress, in the State of New York and the County of Erie. By E. C. W. O'BRIEN, M. D. .... 470

SOCIETY PROCEEDINGS.

Transactions of the Fourth Annual Meeting of the American Association of Obstetricians and Gynecologists .... 475
The Medical Society of Niagara County .... 484

PROGRESS IN MEDICAL SCIENCE.

Medical Jurisprudence .... 487
Medicine .... 489
The Physical Effects of Child Marriage in India .... 491

SELECTION.

A Neuro-Topographical Bust .... 493

NEW INSTRUMENTS.

A Pleura-Trocar .... 495

EDITORIAL.

The Medical Society of the State of New York .... 496
The Students of Niagara University Speak .... 498
Notes and Comment .... 499

PERSONAL.

Dr. A. F. O'Hara.—Dr. Richard A. Heath.—Dr. Wm. C. Krauss.—Dr. Walter Coles .... 501
Dr. Wm. B. Southard.—Dr. William H. Heath.—Dr. Byron Stanton .... 502

Book Reviews .... 502
Books and Pamphlets Received .... 509
Miscellany .... 511

